# Association between alcohol consumption and the risk of ovarian cancer: a meta-analysis of prospective observational studies

**DOI:** 10.1186/s12889-015-1355-8

**Published:** 2015-03-07

**Authors:** Huang Yan-Hong, Li Jing, Luan Hong, Huang Shan-Shan, Li Yan, Li Ju

**Affiliations:** Shenyang Women and Children Health Centre, No. 74, Chongshan Road, Huanggu District, 110032 Shenyang City, Liao Ning China

**Keywords:** Alcohol intake, Ovarian cancer, Meta-analysis

## Abstract

**Background:**

Alcohol consumption has been inconsistently associated with the risk of ovarian cancer. The purpose of this study was to summarize the data from prospective cohort studies on the relationship between alcohol consumption and ovarian cancer using a meta-analytic approach.

**Methods:**

We performed electronic searches of PubMed, Embase, and the Cochrane Library in May 2014 to identify studies that examined the effects of alcohol consumption on the incidence of ovarian cancer. Only prospective cohort studies that reported effect estimates about the incidence of ovarian cancer with 95% confidence intervals (CIs) of alcohol intake were included.

**Results:**

Collectively, we included 13 prospective studies that reported on data from 1,996,841 individuals and included 5,857 cases of ovarian cancer. Alcohol consumption had little to no effect on ovarian cancer incidence when compared to non-drinkers (risk ratio [RR], 1.03; 95% CI, 0.96–1.10; P = 0.473). Similarly, low (RR, 0.96; 95% CI, 0.93–1.00; P = 0.059), moderate (RR, 1.08; 95% CI, 0.92–1.27; P = 0.333), and heavy (RR, 0.99; 95% CI, 0.88–1.12; P = 0.904) alcohol consumption was not associated with the risk of ovarian cancer. Furthermore, subgroup analyses suggested that low alcohol intake was associated with a reduced risk of ovarian cancer whereas heavy alcohol intake was associated with an increased risk of ovarian cancer in multiple subpopulations.

**Conclusions:**

Our study suggests that alcohol intake is not associated with an increased risk of ovarian cancer. Subgroup analyses indicated that alcohol consumption might be associated with the risk of ovarian cancer in specific population or in studies with specific characteristics.

**Electronic supplementary material:**

The online version of this article (doi:10.1186/s12889-015-1355-8) contains supplementary material, which is available to authorized users.

## Background

Alcohol is a commonly consumed beverage that has both favourable and adverse effects on disease morbidity and mortality [1]. Previous meta-analyses have shown that light-to-moderate alcohol consumption is associated with a decreased risk of thyroid cancer [2], Hodgkin lymphoma [3], endometrial cancer [4], and renal cell cancer [5], but it has no significant effects on the risk of breast [6], bladder [7], pancreatic [8], gastric [9], and lung [10] cancers. Additionally, some studies have suggested that heavy alcohol consumption is associated with an increased the risk of gastric, pancreatic, and breast cancers [8,9,11-13]. However, concerns have been raised regarding the accompanied risk of ovarian cancer, which has not been confirmed by previous studies.

The association between alcohol consumption and an increased risk of ovarian cancer was first revealed in the Breast Cancer Detection Demonstration Project [14]. More recently, the risk of ovarian cancer was found to be 47% higher in subjects with moderate alcohol intake (15–30 g/day) in a U.S. cohort [15]. A collaborative analysis of 23 case control studies, three cohort studies, and one pooled analysis did not support an association between alcohol consumption and ovarian cancer risk [16]. However, the conclusions were not consistent between studies, and the data regarding the association between alcohol consumption and ovarian cancer morbidity were both limited and inconclusive. In this study, we performed a systematic review and meta-analysis of cohort studies to evaluate the association between alcohol intake and the incidence of ovarian cancer.

## Methods

### Data sources, search strategy, and selection criteria

This review was conducted and reported according to the Preferred Reporting Items for Systematic Reviews and Meta-Analysis Statement, 2009 (Additional file 1: PRISMA Checklist) [17].

We systematically searched PubMed, Embase, and Cochrane Library electronic databases (from database inception to May 2014) for prospective studies in humans that examined the relationship between alcohol consumption and ovarian cancer; these studies were eligible for inclusion in our study and no restrictions were placed on language or publication status (published or in press). Our core search included the following terms: “ethanol” OR “alcohol” OR “alcoholic beverages” OR “drinking behaviour” OR “alcohol drinking” OR “drink” OR “liquor” OR “ethanol intake” OR “alcohol drink” OR “ethanol drink” AND (“ovarian cancer” OR “ovarian neoplasm” OR “ovarian carcinoma” OR “ovary cancer” OR “ovary neoplasm” OR “ovary carcinoma”) AND (“cohort” OR “cohort studies”). If a site-specific dataset was published more than once, we used the most recent publication. We reviewed the reference lists of the reports, reviews, meta-analyses, and other relevant publications to identify additional pertinent studies. The medical subject heading, methods, population, study design, exposure, and outcome variables of these articles were used to identify relevant studies.

A study was eligible for inclusion if the following criteria were met: (1) the study had a prospective design (prospective cohort or nested prospective case control study), (2) the study investigated the association between alcohol intake and the risk of ovarian cancer, and (3) the authors reported effect estimates (risk ratio [RR] or hazard ratio [HR]) and 95% confidence intervals (CIs) for comparisons between individuals with high alcohol consumption and individuals who did not consume alcohol. We excluded all case–control studies because various confounding factors could have biased the results.

The literature search was independently performed by two authors using a standardized approach. Any inconsistencies were resolved through discussions with the primary author to reach a consensus. We excluded studies that were not published as full reports, which included conference abstracts and letters to the editor.

### Data collection and quality assessment

The following information was collected: the first author or study group name, publication year, country, study design, sample size, ovarian cancer cases, age at baseline, effect estimate, follow-up duration, and covariates in the fully adjusted model. For studies that reported several multivariable adjusted RRs, we selected the effect estimate that was adjusted for the maximum number of potential confounders.

The Newcastle-Ottawa Scale (NOS) [18] was used to evaluate the methodological quality. The NOS is a partially validated comprehensive tool for evaluating the quality of observational studies in meta-analyses [19]. It is based on the following three subscales: selection (4 items), comparability (1 item), and outcome (3 items). A “star system” (range 0–9) was developed for assessment. Two authors independently performed the data extraction and quality assessment. Information was examined and verified independently by an additional author.

### Statistical analysis

We examined the relationship between alcohol intake and risk of ovarian cancer on the basis of the effect estimate (RR or HR) and the 95% CI published in each study. We first used a fixed-effect model to calculate summary RRs and 95% CIs for the different alcohol intake levels and for non-drinkers. We then combined the RRs for drinkers versus non-drinkers by using a random-effect meta-analysis [20,21]. Heterogeneity between studies was investigated by using the Q statistic, and we considered P-values < 0.10 to be indicative of significant heterogeneity [22,23]. Subgroup analyses were conducted for ovarian cancer on the basis of country, type of alcohol consumption, duration of follow-up, adjusted for oral contraceptive use or not, adjusted for hormone replacement therapy or not, adjusted for menopausal status or not, adjusted for smoking status or not, adjusted for body mass index or not, and the NOS score.

We also performed a sensitivity analysis by removing each study from the meta-analysis individually [24]. Several methods were used to check for potential publication bias. Visual inspections of the funnel plots for ovarian cancer were conducted. The Egger [25] and Begg [26] tests were also used to statistically assess publication bias for ovarian cancer. All reported P-values were two-sided and P-values < 0.05 were considered statistically significant for all studies. Statistical analyses were performed using the STATA software (version 12.0; Stata Corporation, College Station, TX, USA).

## Results

**Figure 1:**
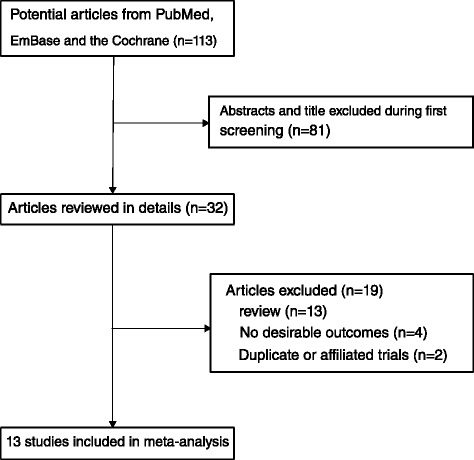
**Flow diagram of the literature search and studies selection process.**

The results of the study selection process are shown in Figure 1. We identified 113 research articles in our initial electronic search, of which 81 were excluded because they were irrelevant. A total of 32 potentially eligible studies were then selected. After detailed evaluations, 13 prospective studies [14,15,27-37] were selected for the final meta-analysis. A manual search of the reference lists of these studies did not yield any new eligible studies. The general characteristics of the included studies are presented in Table 1 and Additional file 2: Table S1. Of the 13 cohort studies that reported data from 1,996,841 individuals, the follow-up period for participants was 5.0–16.5 years and 22,550–1,280,296 individuals were included in each study. Eight studies were conducted in the U.S. [14,15,28-31,33,34], one in the UK [27], one in Sweden [32], one in the Netherlands [35], one in Canada [36], and one in Japan [37]. Study quality was assessed using the NOS [18]. Here, we considered a study with a score ≥ 7 to be of high quality. Overall, two studies [29,31] had a score of 9, five studies [14,27,32,35,36] had a score of 8, three studies [28,33,37] had a score of 7, two studies [15,34] had a score of 6, and one [30] had a score of 5.

**Table 1:** **Baseline characteristic of studies included in the systematic review and meta-analysis**

**Study**	**Country**	**Study design**	**Sample size**	**Cases**	**Age at baseline**	**Effect estimate**	**Follow-up (year)**	**Covariates in fully adjusted model**	**NOS score**
ET Chang 2007 [15]	US	Cohort	90371	253	50.0	RR	8.1	Race, total energy intake, parity, oral contraceptive use, strenuous exercise, and menopausal status/HRT use; stratified by age at baseline	6
NE Allen 2009 [27]	UK	Cohort	1280296	3559	55.9	RR	7.2	Age, region of residence, socioeconomic status, BMI, smoking, physical activity, use of oral contraceptives, and HRT	8
EV Bandera 1997 [28]	US	Cohort	22550	77	50-93	RR	7.0	Age, education, cigarettes/day, years smoking, and total energy intake	7
ER Bertone 2002 (a) [29]	US	Cohort	80195	120	34-59	RR	16.0	Age, parity, age at menarche, menopausal status/postmenopausal HRT used, tubal ligation, and smoking status	9
ER Bertone 2002 (b) [29]	US	Cohort	59538	315	40-65	RR	16.0	Age, parity, age at menarche, menopausal status/postmenopausal HRT used, tubal ligation, and smoking status	9
EE Calle 2002 [30]	US	Cohort	60796	278	50-74	RR	5.0	Calendar year age at menarche, menopausal status at baseline, oral contraceptive use, HRT use among postmenopausal women, parity, BMI, smoking status, physical activity, and energy intake	5
LE Kelemen 2004 [31]	US	Cohort	27205	147	55-69	RR	15.0	Age at menopause, physical activity, postmenopausal HRT, oral contraceptive use, family history of breast cancer, family history of ovarian cancer, known diabetes at baseline, smoking, energy-adjusted intakes of total carotene, vitamin C and vitamin E	9
JV Lacey 2002 [14]	US	Cohort	32885	142	40-93	RR	13.4	Age, menopause type, and oral contraceptive use	8
SC Larsson 2004 [32]	Sweden	Cohort	61103	287	40-74	RR	10.5	Age, BMI, educational level, family history of breast cancer, parity, age at first birth, oral contraceptive use, age at menarche, age at menopause, postmenopausal HRT, fruit and vegetable, lactose, and total energy intake.	8
J Lin 2004 [33]	US	Cohort	32466	104	45-89	RR	8.7	Age, random treatment assignment, BMI, family history of colorectal cancer, history of colorectal polyps, physical activity, cigarette smoking, postmenopausal HRT, and total energy intake	7
B Rockhill 1998 [34]	US	Cohort	91502	52	27-44	RR	6.0	Age at baseline, age at menarche, history of benign breast disease, history of breast cancer in mother and/or sister, height, oral contraceptive history, and parity and age at first birth	6
LJ Schouten 2004 [35]	Netherland	Cohort	62573	214	55-69	RR	9.3	Age, use of oral contraceptives, parity, height, BMI, total energy intake, and current cigarette smoking	8
PD Terry 2003 [36]	Canada	Cohort	49613	223	40-59	RR	16.5	Age in 5-year age groups, treatment allocation, study centre, Quetelet’s index, education level, vigorous physical activity, oral contraceptive use, HRT, parity, age of menarche, and menopausal status	8
E Weiderpass 2012 [37]	Japan	Cohort	45748	86	40-69	HR	16.0	Age, study center, age at menarche, nulliparous, parity, age at first birth, breastfeeding, use of exogenous hormones, menopausal status, height, BMI, smoking status, exposure to second-hand smoke, physical activity, usual sleep duration, family history of cancer	7

**Figure 2:**
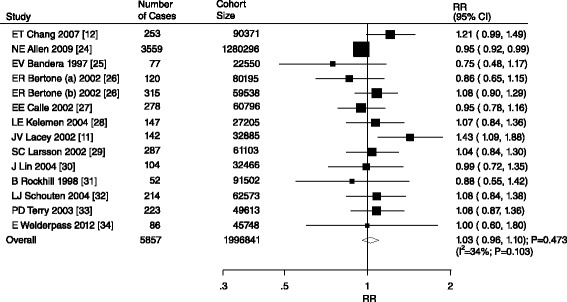
**Relative risk estimates of ovarian cancer for drinker versus non-drinker.**

An association between alcohol consumption and ovarian cancer was reported in a total of 14 cohorts in 13 studies [14,15,27-37]. The summary RR showed that alcohol consumption was not associated with ovarian cancer (RR, 1.03; 95% CI, 0.96–1.10; P = 0.473; Figure 2) but potential evidence of significant heterogeneity was observed (P = 0.103). As a result, a sensitivity analysis was conducted. Sequential exclusion of each individual study from the pooled analysis demonstrated that the results were not affected by the exclusion of any specific study.

**Figure 3:**
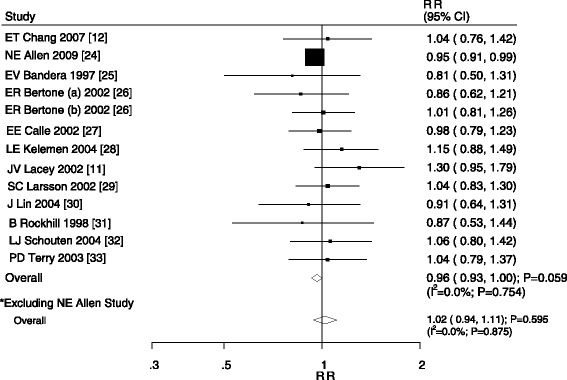
**Relative risk estimates of ovarian cancer for low alcohol consumption versus non-drinker.**

An association between low alcohol intake (<15 g/day) and ovarian cancer was reported in a total of 13 cohorts in 12 studies [14,15,27-36]. We also determined that low alcohol intake was associated with a 4% reduced risk of ovarian cancer, but this association was not statistically significant (RR, 0.96; 95% CI, 0.93–1.00; P = 0.059; Figure 3). Although no evidence of heterogeneity was observed, we also conducted a sensitivity analysis and concluded that low alcohol intake was not associated with a reduced the risk of ovarian cancer (RR, 1.02; 95% CI, 0.94–1.11; P = 0.595; Figure 3).

**Figure 4:**
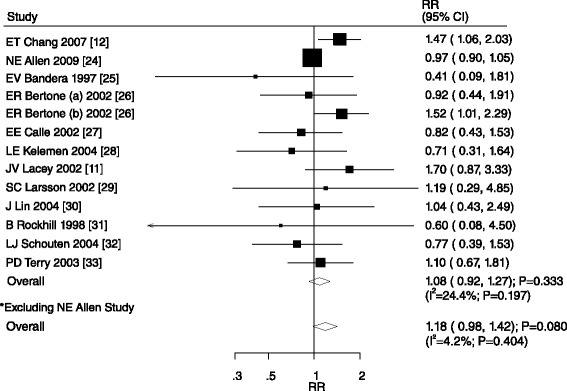
**Relative risk estimates of ovarian cancer for moderate alcohol consumption versus non-drinker.**

An association between moderate alcohol intake (15–30 g/day) and ovarian cancer was reported in a total of 13 cohorts in 12 studies [14,15,27-36]. The results from a pooled analysis indicated that there was no association between moderate alcohol intake and ovarian cancer (RR, 1.08; 95% CI, 0.92–1.27; P = 0.333; Figure 4). Although there was some evidence of heterogeneity across the studies (P = 0.197), a sensitivity analysis indicated that the results were not affected by sequential exclusion of any individual study from the pooled analysis.

**Figure 5:**
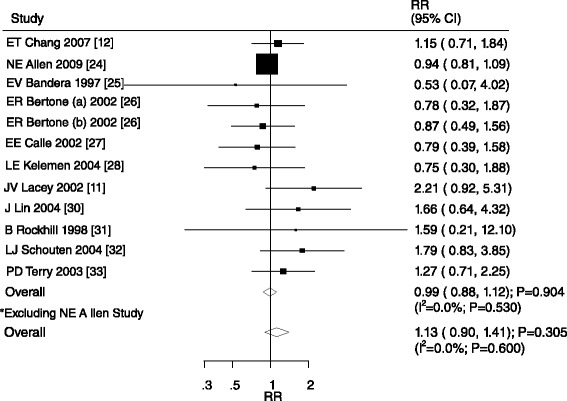
**Relative risk estimates of ovarian cancer for heavy alcohol consumption versus non-drinker.**

A total of 12 cohorts in 11 studies [14,15,27-31,33-37] reported an association between heavy alcohol intake (>30 g/d) and ovarian cancer. Overall, there was no significant association between heavy alcohol intake and ovarian cancer (RR, 0.99; 95% CI, 0.88–1.12; P = 0.904; Figure 5). Although there was no heterogeneity was observed across the studies, we conducted a sensitivity analysis after excluding a study by Allen et al. [27]. These results indicated that heavy alcohol consumption was associated with a 13% increased risk of ovarian cancer. However, the increase was not statistically significant (RR, 1.13; 95% CI, 0.90–1.41; P = 0.305; Figure 5).

Heterogeneity testing revealed P > 0.10 for the incidence of ovarian cancer. We conducted subgroup analyses for ovarian cancer to minimize heterogeneity between the studies and evaluated the association between alcohol intake and the risk of ovarian cancer in specific population or in studies with specific characteristics (Table 2). First, we determined that alcohol consumption was associated with a reduced risk of ovarian cancer if the individuals were from other countries, the study was not adjusted for menopausal status, and if the study was adjusted for smoking status and body mass index. Furthermore, we found that alcohol consumption was associated with an increased risk of ovarian cancer if the study was not adjusted for smoking status. Second, low alcohol intake was associated with a reduced risk of ovarian cancer if the participants were from other countries, the follow-up duration was < 10 years, the study was adjusted for hormone replacement therapy, smoking status, and body mass index, and the study was not adjusted for menopausal status. Third, moderate alcohol consumption was associated with an increased risk of ovarian cancer if the study was adjusted for menopausal status but not for smoking status and body mass index. Finally, heavy alcohol consumption was associated with an increased risk of ovarian cancer if the study was not adjusted for hormone replacement therapy.

**Table 2:** **Subgroup analysis of ovarian cancer for alcohol intake versus nondrinker**

**Category**	**Subgroup**	**Number of cohorts**	**RR and 95% CI**	**P value**	**P value for heterogeneity test**
**Drinker versus nondrinker**	**Country**				
	US	9	1.05 (0.94-1.17)	0.428	0.126
	Other	5	0.96 (0.92-0.99)	0.017	0.597
	**Type of alcohol intake**				
	Wine	2	1.13 (0.92-1.38)	0.254	0.085
	Beer	2	1.01 (0.86-1.18)	0.914	0.250
	Liquor	2	1.02 (0.92-1.14)	0.652	0.789
	**Follow-up duration**				
	More than 10 years	7	1.08 (0.98-1.19)	0.136	0.348
	Less than 10 years	7	0.98 (0.91-1.06)	0.631	0.282
	**Adjusted oral contraceptive**				
	Yes	9	1.05 (0.96-1.16)	0.262	0.038
	No	5	0.98 (0.87-1.12)	0.797	0.506
	**Adjusted hormone replacement therapy**				
	Yes	10	0.98 (0.94-1.03)	0.521	0.373
	No	4	1.05 (0.81-1.37)	0.700	0.064
	**Adjusted menopausal status**				
	Yes	8	1.08 (0.98-1.19)	0.140	0.219
	No	6	0.95 (0.92-0.99)	0.009	0.674
	**Adjusted smoking status**				
	Yes	9	0.96 (0.92-0.99)	0.011	0.716
	No	5	1.14 (1.01-1.30)	0.036	0.285
	**Adjusted body mass index**				
	Yes	6	0.96 (0.92-0.99)	0.010	0.890
	No	8	1.07 (0.96-1.21)	0.226	0.139
	**NOS score**				
	8 or 9	8	1.04 (0.95-1.14)	0.388	0.057
	<8	6	1.01 (0.89-1.15)	0.859	0.368
**Low alcohol intake versus nondrinker**	**Country**				
	US	9	1.01 (0.92-1.12)	0.803	0.665
	Other	4	0.96 (0.92-1.00)	0.033	0.686
	**Follow-up duration**				
	More than 10 years	6	1.06 (0.95-1.18)	0.315	0.595
	Less than 10 years	7	0.95 (0.91-0.99)	0.017	0.957
	**Adjusted oral contraceptive**				
	Yes	9	0.97 (0.93-1.00)	0.083	0.520
	No	4	0.94 (0.80-1.09)	0.396	0.784
	**Adjusted hormone replacement therapy**				
	Yes	9	0.96 (0.92-1.00)	0.039	0.871
	No	4	1.06 (0.87-1.28)	0.584	0.335
	**Adjusted menopausal status**				
	Yes	7	1.03 (0.93-1.13)	0.595	0.753
	No	6	0.95 (0.92-0.99)	0.024	0.680
	**Adjusted smoking status**				
	Yes	8	0.96 (0.92-0.99)	0.024	0.817
	No	5	1.07 (0.94-1.22)	0.331	0.691
	**Adjusted body mass index**				
	Yes	5	0.96 (0.92-0.99)	0.025	0.871
	No	8	1.03 (0.93-1.15)	0.534	0.624
	**NOS score**				
	8 or 9	8	0.98 (0.93-1.03)	0.408	0.391
	<8	5	0.95 (0.82-1.10)	0.526	0.911
**Moderate alcohol intake versus nondrinker**	**Country**				
	US	9	1.20 (0.95-1.52)	0.126	0.296
	Other	4	0.97 (0.90-1.05)	0.440	0.858
	**Follow-up duration**				
	More than 10 years	6	1.24 (0.97-1.60)	0.087	0.496
	Less than 10 years	7	1.02 (0.83-1.25)	0.876	0.213
	**Adjusted oral contraceptive**				
	Yes	9	1.06 (0.89-1.26)	0.537	0.239
	No	4	1.15 (0.77-1.73)	0.500	0.281
	**Adjusted hormone replacement therapy**				
	Yes	9	1.09 (0.92-1.30)	0.307	0.185
	No	4	0.95 (0.51-1.74)	0.859	0.207
	**Adjusted menopausal status**				
	Yes	7	1.31 (1.08-1.59)	0.007	0.538
	No	6	0.96 (0.89-1.04)	0.323	0.785
	**Adjusted smoking status**				
	Yes	8	0.98 (0.89-1.08)	0.662	0.412
	No	5	1.37 (1.07-1.75)	0.012	0.734
	**Adjusted body mass index**				
	Yes	5	0.97 (0.90-1.04)	0.370	0.938
	No	8	1.26 (1.01-1.58)	0.037	0.344
	**NOS score**				
	8 or 9	8	1.05 (0.89-1.23)	0.580	0.300
	<8	5	1.08 (0.73-1.59)	0.713	0.249
**Heavy alcohol intake versus nondrinker**	**Country**				
	US	9	1.04 (0.80-1.35)	0.758	0.605
	Other	3	1.11 (0.79-1.56)	0.539	0.178
	**Follow-up duration**				
	More than 10 years	5	1.07 (0.76-1.51)	0.691	0.339
	Less than 10 years	7	0.98 (0.86-1.12)	0.757	0.517
	**Adjusted oral contraceptive**				
	Yes	8	1.05 (0.87-1.27)	0.591	0.330
	No	4	0.94 (0.62-1.44)	0.783	0.591
	**Adjusted hormone replacement therapy**				
	Yes	8	0.96 (0.85-1.09)	0.536	0.814
	No	4	1.76 (1.03-3.01)	0.038	0.655
	**Adjusted menopausal status**				
	Yes	6	1.08 (0.83-1.40)	0.560	0.446
	No	6	0.97 (0.84-1.11)	0.648	0.452
	**Adjusted smoking status**				
	Yes	8	0.95 (0.83-1.08)	0.436	0.651
	No	4	1.32 (0.94-1.84)	0.104	0.637
	**Adjusted body mass index**				
	Yes	4	1.05 (0.77-1.43)	0.760	0.244
	No	8	1.09 (0.84-1.41)	0.522	0.634
	**NOS score**				
	8 or 9	7	1.04 (0.83-1.30)	0.721	0.277
	<8	5	1.08 (0.76-1.54)	0.654	0.694

**Figure 6:**
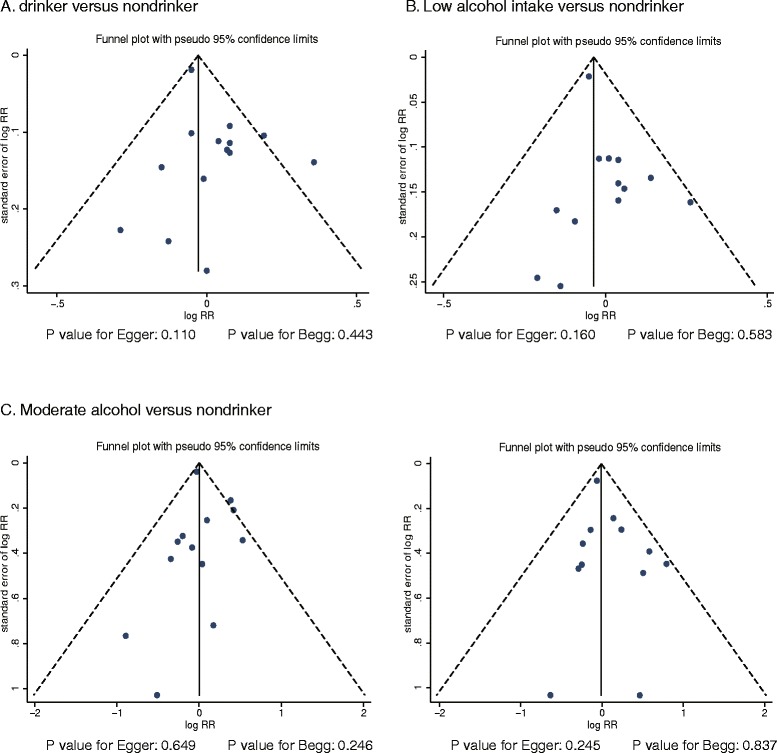
**Funnel plot for ovarian cancer.**

No funnel plot asymmetry was observed for the association between alcohol consumption and ovarian cancer risk (Figure 6). The Egger [25] and Begg [26] tests showed no evidence of publication bias for ovarian cancer (drinkers versus non-drinkers: P-value for the Egger test: 0.110, P-value for the Begg test: 0.443; low alcohol intake versus non-drinkers: P-value for the Egger test: 0.160, P-value for the Begg test: 0.583; moderate alcohol intake versus non-drinkers: P-value for the Egger test: 0.649, P-value for the Begg test: 0.246; heavy alcohol intake versus non-drinkers: P-value for the Egger test: 0.245, P-value for the Begg test: 0.837).

## Discussion

This study was based on prospective studies, and it explored all possible correlations between alcohol consumption and the risk of ovarian cancer. This large and quantitative study included 1,996,841 individuals from 13 prospective cohort studies with a broad range of populations. The findings from our meta-analysis suggest that alcohol consumption has no effect on the incidence of ovarian cancer. Furthermore, low, moderate, and heavy alcohol consumption have no significant effects on the risk of ovarian cancer.

A previous meta-analysis [16] based on observational studies (case control and cohort) suggested that alcohol consumption was not associated with the risk of ovarian cancer. This previous review was inherently limited in that the range of alcohol intake and the cut-off values for the categories differed between studies. Additionally, most studies were case control studies and various confounding factors could have biased the results. Finally, several important factors considered leading risk factors for ovarian cancer were not adjusted, and the subgroup analyses of the previous meta-analysis [16] resulted in no data regarding ovarian cancer caused by these factors. Therefore, we conducted a meta-analysis of prospective cohort studies to evaluate the association between alcohol intake and the risk of ovarian cancer.

Most of our findings were in agreement with a previous published pooled analysis of a cohort study conducted in the U.S. [38]. This large, pooled analysis included 529,638 women and found that alcohol intake was not associated with ovarian cancer risk. Furthermore, Chang et al. [15] suggested that alcohol intake does not affect ovarian cancer risk, but women who consumed at ≥1 glass of wine per day had an increased risk of ovarian cancer. Our current study also indicated that alcohol intake was not associated with the risk of ovarian cancer. However, it might alter the risk of ovarian cancer in specific populations or in studies with specific characteristics. One possible explanation is that alcohol has been shown to block the transport of folate to rapidly proliferating tissues, resulting in impaired haematopoiesis [39]. Furthermore, the data on the different types of alcohol consumed were rarely available. Therefore, we cannot determine whether there is an association between any specific alcohol type and ovarian cancer risk.

We found that there was no significant association between alcohol consumption and the risk of ovarian cancer. However, several studies have reported inconsistent results regarding the association. For example, Chang et al. [15] suggested that moderate alcohol intake was associated with a 47% (95% CI: 1.06–2.03) increase in the risk of ovarian cancer. Similarly, Bertone et al. [28] found that moderate alcohol consumption was associated with a 52% (95% CI: 1.01–2.29) increase in the risk of ovarian cancer. Lacey et al. [14] reported that individuals who consumed alcohol had an increased risk of ovarian cancer compared to non-drinkers (RR, 1.43; 95% CI: 1.09–1.88). Conversely, Allen et al. [27] suggested that alcohol consumption might have a protective effect on the risk of ovarian cancer (RR, 0.95; 95% CI: 0.92–0.99). Moreover, low alcohol intake was associated with a reduced risk of ovarian cancer (RR, 0.95; 95% CI: 0.91–0.99). The reasons for this discrepancy could be the following: (1) the age at baseline in individual studies might be an important confounding factor between alcohol intake and the risk of ovarian cancer; (2) alcohol intake might have a favourable effect by decreasing follicle stimulating and luteinizing hormones and gonadotropin levels, which might have an important effect on the risk of ovarian cancer.

Subgroup analyses suggested that alcohol consumption was associated with a reduction in ovarian cancer if the individuals were from other countries, the study was not adjusted for menopausal status, and if the study was adjusted for smoking status and body mass index. Specifically, low alcohol intake was associated with a decreased the risk of ovarian cancer if the participants were from other countries, the follow-up duration was < 10 years, the study was adjusted for hormone replacement therapy, smoking status, and body mass index, and if the study was not adjusted for menopausal status. In contrast, we determined that alcohol consumption was associated with an increased risk of ovarian cancer if the study was not adjusted for smoking status. Moderate alcohol intake was associated with an increased risk of ovarian cancer if the study was adjusted for menopausal status and if the study was not adjusted for smoking status and body mass index. Finally, heavy alcohol intake was associated with an increased risk of ovarian cancer if the study was not adjusted for hormone replacement therapy.

Our data may be explained by the fact that were multiple interrelations between alcohol intake and country, or by the duration of the follow-up, menopausal status, smoking status, and body mass index. However, we could not determine the effects of the confounding factors on the risk of ovarian cancer because few studies were stratified by these important factors. The duration of alcohol intake was another important factor that was rarely reported in these studies. Therefore, we provided a relative result and a synthetic and comprehensive review.

The limitations of our study are the following: (1) the adjusted models differ between studies and these factors might play an important role in the development of ovarian cancer, (2) publication bias in a meta-analysis of published studies is an inevitable problem, and (3) the analysis was performed using pooled data because individual data were not available, which restricted us from performing a more detailed analysis in order to obtain more comprehensive results.

## Conclusions

The results of this study suggest that alcohol consumption is not associated with ovarian cancer risk. Subgroup analyses indicated that low or moderate alcohol consumption could be associated with the risk of ovarian cancer in specific populations or in studies with specific characteristics. Future studies should focus on specific populations for primary analysis of ovarian cancer prevention.

## Additional files

Additional file 1:
**PRISMA Checklist.**
Additional file 2: Table S1. Relative risk of difference alcohol intake categories versus the nondrinker and the risk of ovarian cancer.
