# Supplementary material for: Association between alcohol consumption and the risk of ovarian cancer: a meta-analysis of prospective observational studies
Source: BMC Public Health. 2015 Mar 7;15:223. doi: 10.1186/s12889-015-1355-8 (PMC4415339; doi:10.1186/s12889-015-1355-8)
Supplement: Additional file 2: Table S1. — Relative risk of difference alcohol intake categories versus the nondrinker and the risk of ovarian cancer. [file 12889_2015_1355_MOESM2_ESM.doc]

Table S1. Relative risk of difference alcohol intake categories versus the nondrinker and the risk of ovarian cancer.

| **Study** | **Drinker** | **Low alcohol intake** | **Moderate alcohol intake** | **Heavy alcohol intake** |
| --- | --- | --- | --- | --- |
| ET Chang 2007 [15] | 1.21 (0.99-1.49) | 1.04 (0.76-1.42) | 1.47 (1.06-2.03) | 1.15 (0.71-1.84) |
| NE Allen 2009 [27] | 0.95 (0.92-0.99) | 0.95 (0.91-0.99) | 0.97 (0.90-1.05) | 0.94 (0.81-1.09) |
| EV Bandera 1997 [28] | 0.75 (0.48-1.17) | 0.81 (0.50-1.31) | 0.41 (0.09-1.81) | 0.53 (0.07-4.02) |
| ER Bertone 2002 (a) [29] | 0.86 (0.65-1.15) | 0.86 (0.62-1.21) | 0.92 (0.44-1.91) | 0.78 (0.32-1.87) |
| ER Bertone 2002 (b) [29] | 1.08 (0.90-1.29) | 1.01 (0.81-1.26) | 1.52 (1.01-2.29) | 0.87 (0.49-1.56) |
| EE Calle 2002 [30] | 0.95 (0.78-1.16) | 0.98 (0.79-1.23) | 0.82 (0.43-1.53) | 0.79 (0.39-1.58) |
| LE Kelemen 2004 [31] | 1.07 (0.84-1.36) | 1.15 (0.88-1.49) | 0.71 (0.31-1.64) | 0.75 (0.30-1.88) |
| JV Lacey 2002 [14] | 1.43 (1.09-1.88) | 1.30 (0.95-1.79) | 1.70 (0.87-3.33) | 2.21 (0.92-5.31) |
| SC Larsson 2004 [32] | 1.04 (0.84-1.30) | 1.04 (0.83-1.30) | 1.19 (0.29-4.85) | - |
| J Lin 2004 [33] | 0.99 (0.72-1.35) | 0.91 (0.64-1.31) | 1.04 (0.43-2.49) | 1.66 (0.64-4.32) |
| B Rockhill 1998 [34] | 0.88 (0.55-1.42) | 0.87 (0.53-1.44) | 0.60 (0.08-4.50) | 1.59 (0.21-12.10) |
| LJ Schouten 2004 [35] | 1.08 (0.84-1.38) | 1.06 (0.80-1.42) | 0.77 (0.39-1.53) | 1.79 (0.83-3.85) |
| PD Terry 2003 [36] | 1.08 (0.87-1.36) | 1.04 (0.79-1.37) | 1.10 (0.67-1.81) | 1.27 (0.71-2.25) |
| E Weiderpass 2012 [37] | 1.00 (0.60-1.80) | - | - | - |
